# Antimicrobial resistance of *Neisseria gonorrhoeae* isolates in south-west Germany, 2004 to 2015: increasing minimal inhibitory concentrations of tetracycline but no resistance to third-generation cephalosporins

**DOI:** 10.2807/1560-7917.ES.2016.21.36.30335

**Published:** 2016-09-08

**Authors:** Thomas Regnath, Thomas Mertes, Ralf Ignatius

**Affiliations:** 1 Laboratory Enders and Partners, Stuttgart, Germany; 2 MVZ of Laboratory Medicine and Microbiology Koblenz-Mittelrhein, Koblenz, Germany; 3 Department of Microbiology and Hygiene, Charité – Universitätsmedizin Berlin, Berlin, Germany; 4 Laboratory 28, Berlin, Germany; 5 Affiliations at the time of submission: 1,3; current affiliations: 3,4

**Keywords:** gonorrhoea, *Neisseria gonorrhoeae*, emerging or re-emerging diseases, antimicrobial resistance, sexually transmitted infections

## Abstract

Increasing antimicrobial resistance of *Neisseria gonorrhoeae*, particularly to third-generation cephalosporins, has been reported in many countries. We examined the susceptibility (determined by Etest and evaluated using the breakpoints of the European Committee on Antimicrobial Susceptibility Testing) of 434 *N. gonorrhoeae* isolates collected from 107 female and 327 male patients in Stuttgart, south-west Germany, between 2004 and 2015. During the study period, high proportions of isolates were resistant to ciprofloxacin (70.3%), tetracycline (48.4%; increasing from 27.5% in 2004/2005 to 57.7% in 2014/2015; p = 0.0002) and penicillin (25.6%). The proportion of isolates resistant to azithromycin was low (5.5%) but tended to increase (p = 0.08). No resistance and stable minimum inhibitory concentrations were found for cefixime, ceftriaxone, and spectinomycin. High-level resistance was found for ciprofloxacin (39.6%) and tetracycline (20.0%) but not for azithromycin; 16.3% of the isolates produced betalactamase. Thus, cephalosporins can still be used for the treatment of gonorrhoea in the study area. To avoid further increasing resistance to azithromycin, its usage should be limited to patients allergic to cephalosporins, or (in combination with cephalosporins) to patients for whom no susceptibility testing could be performed or those co-infected with chlamydiae.

## Introduction

Numbers of gonorrhoea cases have increased, and the World Health Organization has estimated 106 million new cases in adults worldwide for 2008, which was 21% higher than numbers for 2005 [1]. At the same time, rising rates of antimicrobial resistance of its causative agent, *Neisseria gonorrhoeae*, have been reported in many parts of the world including Europe, even against the third-generation cephalosporins, cefixime and ceftriaxone [2,3]. For this reason, cefixime alone is no longer recommended as single-drug treatment for gonorrhoea in Europe or the United States [4,5].

The European Gonococcal Antimicrobial Surveillance Programme (EURO-GASP) was established by 12 European countries in 2004 in response to the emerging antimicrobial resistance of *N. gonorrhoeae*, as part of the European Surveillance of Sexually Transmitted Infections Project [6]. In 2016, EURO-GASP has participation from laboratories from 21 European Union/European Economic Area countries, which regularly report gonorrhoea susceptibility testing results and epidemiological surveillance data, and submit gonococcal isolates for centralised testing or participate in decentralised testing. Since 2009, EURO-GASP has been coordinated by the European Centre for Disease Prevention and Control (ECDC). Resistance data have been published regularly and in a timely manner [7,8], but the numbers of isolates tested per country are relatively low (between 10 and 251 in 2011 [8]) and therefore most likely not representative of the epidemiological situation of the individual countries.

As cases of gonorrhoea or antimicrobial resistance patterns of *N. gonorrhoeae* isolates are not subject to reporting in Germany, data regarding current antimicrobial susceptibility and its development over time are scarce. Only three individual studies have addressed this issue in the past 10 years. Abraham et al. analysed 50 isolates collected between 2001 and 2010 in Dresden, Saxony [9], while Horn and colleagues have reported the results of a nationwide surveillance study conducted by the Paul-Ehrlich-Society of Chemotherapy in 2010/2011 in which 213 isolates submitted by 23 laboratories were analysed [10]. Additionally, minimum inhibitory concentrations (MICs) of selected antibiotics for 65 *N. gonorrhoeae* isolates collected in 2004/2005 in southern Germany have been reported [11]. None of these studies has reported cephalosporin-resistant *N. gonorrhoeae* isolates. Data from EURO-GASP, however, have provided evidence for the presence of cephalosporin-resistant *N. gonorrhoeae* isolates in Germany, too [7,8]; in fact, an Austrian patient with a cefixime-resistant *N. gonorrhoeae* isolate acquired his infection in Munich, south Germany [12].

To gain more information on the antimicrobial susceptibility of *N. gonorrhoeae* in Germany and elucidate possible changes in antimicrobial resistance occurring over time, we analysed the susceptibility patterns of all *N. gonorrhoeae* isolates identified and tested in our laboratory between 2004 and 2015 (n = 434). Since age and sex have been identified as risk factors for harbouring antimicrobial-resistant *N. gonorrhoeae* isolates [13,14], we additionally analysed our data regarding these parameters. Unfortunately, the study design chosen did not provide information regarding other possible risk factors, e.g. working as professional sex worker or being a man who has sex with men (MSM).

## Methods

### Bacterial isolates 

Between July 2004 and March 2015, 434 bacterial isolates were grown from swabs obtained from patients aged 16 years and over living in the greater Stuttgart area (a radius of around 50 km from Stuttgart city centre). Although difficult to estimate because *N. gonorrhoeae* is not mandatorily notifiable, the number of isolates tested may correspond to 30–70% of all isolates detected in that area within the period of time indicated. The samples were mainly submitted by private practitioners (primarily urologists, internists, dermatologists, and gynaecologists). For 284 (65.4%) patients, further samples (swabs or urine) had been submitted for the detection of *Chlamydia trachomatis* by PCR. Based on these laboratory results, 36 (12.7%) patients were co-infected with *C. trachomatis* while 248 (87.3%) were *C. trachomatis*-negative.

Gram-negative, oxidase-positive diplococci were identified biochemically as *N. gonorrhoeae* by using the API or the Vitek 2 system (both bioMerieux, Marcy-l Etoile, France). Isolates were subjected immediately to antimicrobial susceptibility testing and subsequently stored at –70 °C for future analyses.

### Susceptibility testing

Briefly, bacterial inoculums were prepared in sterile saline at 0.5 McFarland standard and tested against the antibiotics indicated by using the Etest system (AB Biodisk, Solna, Sweden) on Mueller-Hinton chocolate agar, except for testing of azithromycin, for which a chocolate GC II agar with IsoVitaleX was used (both, BD Diagnostic Systems, Heidelberg, Germany) [11]. Plates were incubated at 35–36.5 °C and 5% CO_2_ for 20–24 hours. Betalactamase production was assessed by using nitrocefin disks (BD Diagnostic Systems). The ATCC *N. gonorrhoeae* strain 49226 was used as an internal control. Susceptibility was interpreted according to the European Committee on Antimicrobial Susceptibility Testing (EUCAST) and Clinical and Laboratory Standards Institute (CLSI) breakpoints [15,16].

### Statistical analyses

Data were statistically analysed by using GraphPad Prism version 6.0a for Mac OS X. Data on antibiotic resistance, patients’ sex, or specialisation of referring practitioners were analysed using the two-tailed Fisher’s exact test. Data regarding age were analysed using the Mann–Whitney U-test. To elucidate potential changes in the antibiotic susceptibility in *N. gonorhoeae* isolates over time, data also were analysed for two different periods: July 2004 to December 2009 and January 2010 to March 2015, as well as for two-year periods. Differences were considered statistically significant at p < 0.05. Trends were defined as p-values between 0.05 and 0.1.

## Results

### Resistance patterns 

The median age of the patients was 33 years (range: 16–76), 107 (24.7%) were female and 327 (75.3%) were male. Analysis of antimicrobial susceptibility of the isolates to standard antibiotics revealed high proportions of intermediate or resistant isolates for penicillin (90.6%), ciprofloxacin (70.5%), and tetracycline (68.9%) with lower proportions for azithromycin (26.5%; Table 1). No resistance to cefixime, ceftriaxone, or spectinomycin was detected. Thirty-one isolates (7.1%) were susceptible to all antimicrobials tested.

**Table 1:** Antimicrobial activities of selected antibiotics against *Neisseria gonorrhoeae* isolates, Stuttgart, 2004–2015 (n=434)

	MIC (mg/L)			% of isolates (S/I/R*)	
**Antimicrobial agent**	**MIC_50_**	**MIC_90_**	**Range**	**EUCAST** [8]	**CLSI** [9]
Penicillin G	0.5	8	0.002 to >32	9.4/65.0/25.6	9.4/65.0/25.6
Cefixime	0.016	0.03	<0.016 to 0.125	100/NM/0	100/ NM /0
Ceftriaxone^a^	0.008	0.03	<0.002 to 0.125	100/NM/0	100/NM/0
Azithromycin	0.25	0.5	0.016 to 128	73.5/21.0/5.5	n.d.
Ciprofloxacin	2	32	<0.002 to >32	29.5/0.2/70.3	29.7/5.8/64.5
Tetracycline	2	32	0.016 to >256	31.1/20.5/48.4	17.3/34.3/48.4
Spectinomycin	8	16	1 to 32	100/NM/0	100/NM/0

CLSI: Clinical and Laboratory Standards Institute; EUCAST: European Committee on Antimicrobial Susceptibility Testing; I: intermediate; MIC: minimum inhibitory concentrations; n.d.: not determined (no breakpoints available); NM: no MICs for intermediate susceptibility defined; R: resistant; S: susceptible.

^a^ Data available for 432 isolates.

High-level plasmid-mediated resistance to tetracycline (≥ 16 mg/L) was seen in 87 (20.0%) isolates whereas 172 (39.6%) isolates expressed high-level resistance to ciprofloxacin (≥ 4 mg/L). Seventy of 430 (16.3%) for which data on betalactamase production was available were betalactamase positive and thus expressed high-level plasmid-mediated resistance to penicillin. High-level resistance to azithromycin (≥ 256 mg/L) was not detected.

*N. gonorrhoeae* isolates from female patients (n = 107) were more resistant to penicillin (33.6%, 95% CI: 25.4–43.0% vs 23.2%, 95% CI: 19.0–28.1%; p = 0.041) and ciprofloxacin (78.5%, 95% CI: 69.7–85.3% vs 68.2% 95% CI: 63.0–73.0; p = 0.0499) than isolates from male patients (n = 327). There was no difference regarding resistance to azithromycin or tetracycline (data not shown). Patients older than 25 years (n = 317) and those aged 25 years or younger (n = 117) did not differ regarding resistance to penicillin (p = 0.536), azithromycin (p = 0.481), ciprofloxacin (p = 0.478) or tetracycline (p = 0.450).

### Comparison of antimicrobial resistance 2004–2009 vs 2010–2015

**Figure 1:**
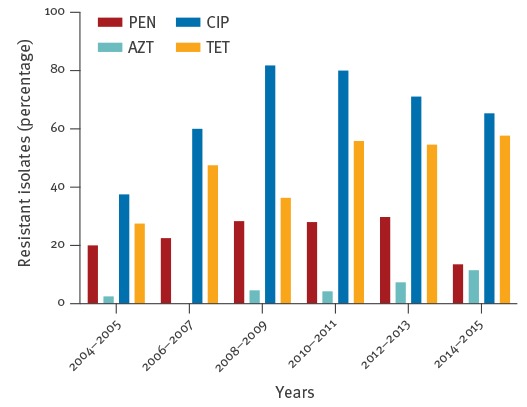
Percentages of *Neisseria gonorrhoeae* isolates resistant to penicillin, azithromycin, ciprofloxacin, or tetracycline, south-west Germany, 2004–2015

AZT: azithromoycin; CIP: ciprofloxacin; PEN: penicillin; TET: tetracycline.

Demographic data of the two subpopulations, patients whose isolates were analysed in 2004–2009 and those whose isolates were analysed 2010–2015, were comparable (Table 2). Applying the EUCAST definitions for resistance [15], the proportion of isolates resistant to tetracycline significantly increased and in fact almost doubled (2004–2009, 30.4%; 2010–2015, 55.6%; Table 3). There also was a trend (p = 0.084) towards increasing resistance to azithromycin (2004–2009, 3.0%; 2010–2015, 7.1%; Table 3) while the resistance to penicillin or ciprofloxacin did not change significantly within the study period. A more detailed analysis by plotting the data for two-year periods confirmed this overall pattern for the whole study period (tetracycline, p = 0.006; penicillin, p = 0.411; ciprofloxacin, p = 0.844; azithromycin, p = 0.133; Figure 1); notably, resistance to ciprofloxacin increased between 2004/05 and 2008/09 but decreased thereafter (p < 0.0001, each).

**Table 2:** Demographic data from *Neisseria gonorrhoeae*-positive patients, south-west Germany, 2004–2009 vs 2010–2015 (n=414)

Characteristics	July 2004 to December 2009	January 2010 to March 2015	p-value
Number of isolates	168	266	
Sex			0.909
Female	42 (25.0%)	65 (24.4%)	
Male	126 (75.0%)	201 (75.6)	
Age in years (median; range)	32; 17-68	34; 16-76	0.718
Specialisation of senders			
Urology	78 (47.0%)	149 (56.0%)	0.076
Gynaecology	37 (22.3%)	61 (22.9%)	0.906
Internal medicine	20 (12.0%)	22 (8.3%)	0.235
Dermatology	16 (9.6%)	20 (7.5%)	0.479
Others or no specialisation	15 (9.0%)	14 (5.3%)	0.167

**Table 3:** Resistance of *Neisseria gonorrhoeae* isolates, south-west Germany, 2004–2009 vs 2010–2015 (n=434)

	2004–2009 (n=168)		2010–2015 (n=266)		
**Antibiotic agent**	**No**	**% (95% CI)** ^a^	**No**	**% (95% CI)**	**p**
Penicillin G (>1 mg/L)	42	25.0 (19.0-32.1)	69	25.9 (21.0-31.5)	0.910
Azithromycin (>0.5 mg/L)	5	3.0 (1.1-7.0)	19	7.1 (4.6-10.9)	0.084
Ciprofloxacin (>0.06 mg/L)	111	66.1 (58.6-72.8)	194	72.9 (67.3-77.9)	0.133
Tetracycline (>1 mg/L)	62	36.9 (30.0-44.4)	148	55.6 (49.6-61.5)	0.0002

CI: confidence interval.

^a^ Percentage of resistant isolates defined by European Committee on Antimicrobial Susceptibility Testing (EUCAST) breakpoints plus 95% CI.

**Figure 2:**
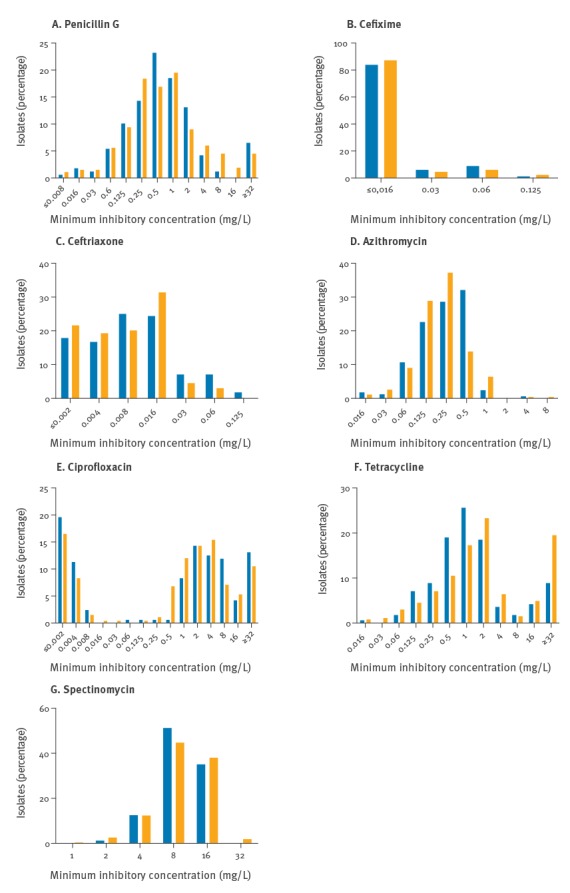
Antimicrobial susceptibility of *Neisseria gonorrhoeae* isolates to (A) penicillin G, (B) cefixime, (C) ceftriaxone, (D) azithromycin, (E) ciprofloxacin, (F) tetracycline and (G) spectinomycin, south-west Germany, July 2004 to December 2015 (n=168) vs January 2010 to March 2015 (n=266)

Analysis of the individual MICs of the seven antibiotics tested revealed some minor changes for most antibiotics (Figure 2). In contrast, proportions of isolates with MICs of 0.5 or 1 mg/L to tetracycline decreased considerably while those of isolates with MICs of 2, 4 or ≥32 mg/L increased (Figure 2F). Likewise, the increase in isolates with MICs > 0.5 mg/L to azithromycin became evident but the proportions of isolates with MICs of 0.125 and 0.25 mg/L were rather higher in the second period than before (Figure 2D). The unique pattern of ciprofloxacin revealed two groups of isolates, i.e. one expressing very low MICs and the other one with MICs ≥ 0.5 mg/L while there were almost no isolates with MICs at the level of the EUCAST breakpoint concentration, 0.06 mg/L, or slightly higher or lower (Figure 2E). Notably, there was a stable pattern for both cefixime and ceftriaxone, excluding also potential shifts of MICs at lower concentrations (Figure 2B and C), which might go undetected when only the breakpoints are considered. 

Blue bars (n=168) represent isolates from July 2004 to December 2009. Yellow bars (n=266) represent isolates from January 2010 to March 2015.

## Discussion

Although we were unable to detect cephalosporin-resistant *N. gonorrhoeae* isolates within the study period, cefixime resistance has been reported for many European countries including Germany [8]. Thus, our data, although in line with two recently published German studies [9,10], cannot be considered representative for the situation in Germany, but they indicate that the true overall cefixime resistance rate in Germany may be considerably lower than the 10.2% reported for 2011 by EURO-GASP [8]. In fact, all laboratory results may strongly depend on the area where the samples were collected and the numbers of patients included from high-risk groups. Our key finding in this context, however, is the absence of an increase in MICs to cephalosporins from the period 2004–2009 to the period 2010–2015 (Figure 1). Thus, a possible rise in cephalosporin-resistance in the future may not be the result of previous and current (mis-)usage of cefixime in the local population, but rather due to the introduction of new resistant strains into that population.

The EUCAST breakpoint of cefixime may be too high, as *N. gonorrhoeae* isolates with this or lower MICs have been described as the cause of infections that did not resolve under cefixime standard therapy [8]. Considering a breakpoint of 0.06 mg/L, seven isolates (1.6%) tested by us with a MIC of 0.125 mg/L were resistant to cefixime.

We detected azithromycin resistance in 5.5% of the isolates and none of the isolates expressed high-level resistance; both findings correspond to the data recently published by Horn et al. [10]. In contrast, an azithromycin resistance rate of 0.9% has been reported for Germany by EURO-GASP [8]. Notably, we observed a strong trend towards more isolates with MICs > 0.5 mg/L of azithromycin from the period 2004­–2009 to 2010–2015 (p = 0.084), although in Europe, resistance significantly decreased between 2009 and 2011 [8]. Still, increasing azithromycin high-level resistance of *N. gonorrhoeae* isolates and a concomitant decline in isolates expressing decreased susceptibility have been shown for Scotland from 2004 to 2007 [17] from where it appears to have spread to England and Wales [18]. Only single high-level resistant isolates have been shown for other European countries, including Italy [19], Sweden [20], France [21] and Ireland [22]. To prevent further increasing resistance to this valuable drug, azithromycin usage should be limited to patients who need to be treated with this drug, i.e. patients with allergy to cephalosporins or patients with simultaneous chlamydial infection (in which case azithromycin should be given together with cephalosporins) and those for whom no drug susceptibility testing of the causative agents can be performed.

The highest resistance rate was observed for ciprofloxacin (70.3%). This is in accordance with the data published by Horn et al. [10] while resistance rates of around 50% have been reported by others for Germany [8,9]. Resistance rates for ciprofloxacin are also high in most other European countries [8], and this might be a sign of a still high fluoroquinolone usage by practitioners for the empirical treatment of gonorrhoeae, as ciprofloxacin resistance of *N. gonorrhoeae* has been shown to decline after cessation of ciprofloxacin usage [23]. It remains unclear at the moment, however, whether the observed increase and decrease of ciprofloxacin resistance correlates with changes in ciprofloxacin usage within the study period. In contrast to the other antimicrobials tested, MICs to ciprofloxacin showed a bimodal distribution similar to what has been shown for *N. gonorrhoeae* isolates from Japan [24].

Tetracycline resistance also was high (as previously observed by others [10]), and increased significantly within the study period (from 27.5% in 2004/2005 to 57.7% in 2014/2015). This might reflect its wide usage. Doxycyline was the second most commonly prescribed antimicrobial drug in German outpatient departments in 2011 [25]. When we compared the antimicrobial susceptibility for 2004–2009 vs 2010–2015, considerably more isolates were included in the latter than in the former period, although the second period was three months shorter than the earlier one. Even if it must remain speculative at the moment, this finding might indicate a true increase in cases of gonorrhoea in the past years, similar to what has been reported for England and Sweden [26,27], and thus may correspond to the trend reported for syphilis in Germany [28]. A reintroduction of the previous obligation to report all cases of gonorrhoea in Germany, currently under discussion, may answer this question in the future. An increasing gonorrhoea incidence, however, also might at least partially result from the previously discussed ciprofloxacin usage for empirical treatment of the infection in the presence of high resistance rates to this drug [29].

We did not detect spectinomycin resistance. This might be due to its long-lasting non-usage, since its usage leads to increasing resistance rates [30]. Thus, this drug could potentially be used for the empirical treatment of gonorrhoea. Known limitations, however, are its unavailability in many European countries including Germany and its reduced effectiveness in the treatment of pharyngeal gonorrhoea [31].

In contrast to previous publications [13,14], we did not observe age-related differences in susceptibility rates, and isolates from female patients showed higher resistance rates than those from male patients. This might be due to differences in study populations. One limitation of our study in this context is the fact that we do not have information regarding patients who may belong to high-risk populations. For instance, it has been shown that *N. gonorrhoeae* isolates from MSM are more resistant to antimicrobials than those collected from men who have sex with women [32,33]. Likewise, infected commercial sex workers are more likely to harbour resistant *N. gonorrhoeae* isolates than other patients [32,34]. In contrast, a recent EURO-GASP study has demonstrated decreasing cefixime MICs for *N. gonorrhoeae* of MSM for the period 2009–2011, and highest MICs for isolates from men who have sex with women [13]. Furthermore, around 75% of the bacterial isolates analysed in the present study were collected from men; thus, our data may be more valid for male than for female patients.

In conclusion, the current resistance situation of *N. gonorrhoeae* isolates in south-west Germany may be less dramatic than in other parts of Germany or other European countries. High resistance rates to some antimicrobials and changes of susceptibility over time, however, call for a more stringent monitoring system, e.g. the obligation of every laboratory to report all *N. gonorrhoeae* isolates together with their susceptibility testing results.
